# Genome-wide unbalanced expression bias and expression level dominance toward *Brassica oleracea* in artificially synthesized intergeneric hybrids of *Raphanobrassica*

**DOI:** 10.1038/s41438-021-00672-2

**Published:** 2021-12-01

**Authors:** Libin Zhang, Jianjie He, Hongsheng He, Jiangsheng Wu, Maoteng Li

**Affiliations:** 1 College of Life Science and Technology, Huazhong University of Science and Technology, Wuhan, 430074 China; 2 National Key Laboratory of Crop Genetic Improvement, Huazhong Agricultural University, Wuhan, 430070 China

**Keywords:** Plant hybridization, Synthetic organisms, RNA sequencing

## Abstract

*Raphanobrassica* (R^r^R^r^C^r^C^r^, 2*n* = 4*x* = 36), which is generated by distant hybridization between the maternal parent *Raphanus sativus* (R^s^R^s^, 2n = 2x = 18) and the paternal parent *Brassica oleracea* (C°C°, 2*n* = 2*x* = 18), displays intermediate silique phenotypes compared to diploid progenitors. However, the hybrid shares much more similarities in silique phenotypes with those of *B. oleracea* than those of *R. sativus*. Strikingly, the silique of *Raphanobrassica* is obviously split into two parts. To investigate the gene expression patterns behind these phenomena, transcriptome analysis was performed on the upper, middle, and lower sections of pods (RCsiu, RCsim, and RCsil), seeds in the upper and lower sections of siliques (RCseu and RCsel) from *Raphanobrassica*, whole pods (Rsi and Csi) and all seeds in the siliques (Rse and Cse) from *R. sativus* and *B. oleracea*. Transcriptome shock was observed in all five aforementioned tissues of *Raphanobrassica*. Genome-wide unbalanced biased expression and expression level dominance were also discovered, and both of them were toward *B. oleracea* in *Raphanobrassica*, which is consistent with the observed phenotypes. The present results reveal the global gene expression patterns of different sections of siliques of *Raphanobrassica*, pods, and seeds of *B. oleracea* and *R. sativus*, unraveling the tight correlation between global gene expression patterns and phenotypes of the hybrid and its parents.

## Introduction

Hybridization provides a way of interspecific or intergeneric genome transfer for incorporating preferable traits from the parents to the progeny. Allopolyploid hybrids generated from interspecific or intergeneric hybridization have provided abundant genetic resources for molecular biology research and crop breeding. Many crops, such as wheat^1^, cotton^2^, rapeseed^3^, and Chinese cabbage^4^, are derived from hybridization between different species. However, polyploidization might induce several important changes at the genetic, gene expression, and epigenetic levels^5–10^. In recent decades, many efforts have been made to produce synthetic allopolyploid species, such as *Arabidopsis*^11,12^, *Brassica*^13,14^, *Oryza*^15^, *Nicotiana*^16^, *Triticum*^17^ and *Gossypium*^18^. Among them, *Brassica* species are one of the model systems to study crop allopolyploidization^19,20^. For example, miRNA expression patterns were explored between *B. napus*, *B. rapa,* and their F1 hybrids^21^. However, the genomic instability and the difficulty of the production of distant hybrids impede a better understanding of global expression patterns and/or regulatory mechanisms of gene expression during hybridization.

Allopolyploids often show genome-wide expression level dominance and/or homoeolog expression bias, namely, the hybrid is more similar and/or biases to one parent than the other^22^. Such expression dominance and/or bias phenomena have been reported in species such as *B. napus*^23^, *P. hesperium*^24^, *G. hirsutum*^25,26^, and *T. turgidum*^27^. One representative example has been recently reported in newly resynthesized *B. napus* allopolyploids, in which genome-wide expression bias and dominance are both toward the A-genome^13^. It has also been revealed that a high proportion of genes in *Triticum turgidum*, which has heterosis, exhibit parental expression level dominance in the hybrid between *T. turgidum* (AABB) and *Aegilops tauschii* (DD)^17^. Although plant polyploidy has potential advantages in growth vigor (heterosis), allopolyploidization usually induces “genomic shock” due to imbalanced and antagonistic gene expression in polyploids^28^.

Although recent research on polyploid hybrid plants has exploded, the majority of the previous reports are based on interspecific and/or intrageneric species. Some studies have also focused on intergeneric hybridizations within the *Brassicaceae* family in the last few years^29,30^. Nevertheless, the global gene expression patterns of different intergeneric hybrids within the *Brassicaceae* family have scarcely been reported. In our group, a synthetic intergeneric hybrid *Raphanobrassica* (R^r^R^r^C^r^C^r^, 2*n* = 36) was constructed by hybridization between *R. sativus* and *B. alboglabra* Bailey, which has high fertility and resistance to clubroot disease and beet cyst nematode effectiveness^31–33^. The newly synthesized hybrid provides a perfect model to study the plant polyploidy generated by crossing distinct important vegetables. In this study, we aimed to explore the global gene expression patterns of *Raphanobrassica* and its parents, determine the relationships between the gene expression and the silique phenotypes in which the hybrid shows intermediate traits compared to its diploid progenitors and shares many more similarities with *B. oleracea*. RNA sequencing was performed, and transcriptome shock was observed in RCsiu, RCsim, RCsil, RCseu, and RCsel of *Raphanobrassica*. The global gene expression patterns of the five tissues of the hybrid were much more similar to those of *B. oleracea* than those of *R. sativus*. The five tissues also showed genome-wide unbalanced biased expression and expression level dominance toward *B. oleracea*.

## Results

### The silique phenotypes of *Raphanobrassica* and its diploid progenitors

**Fig. 1:**
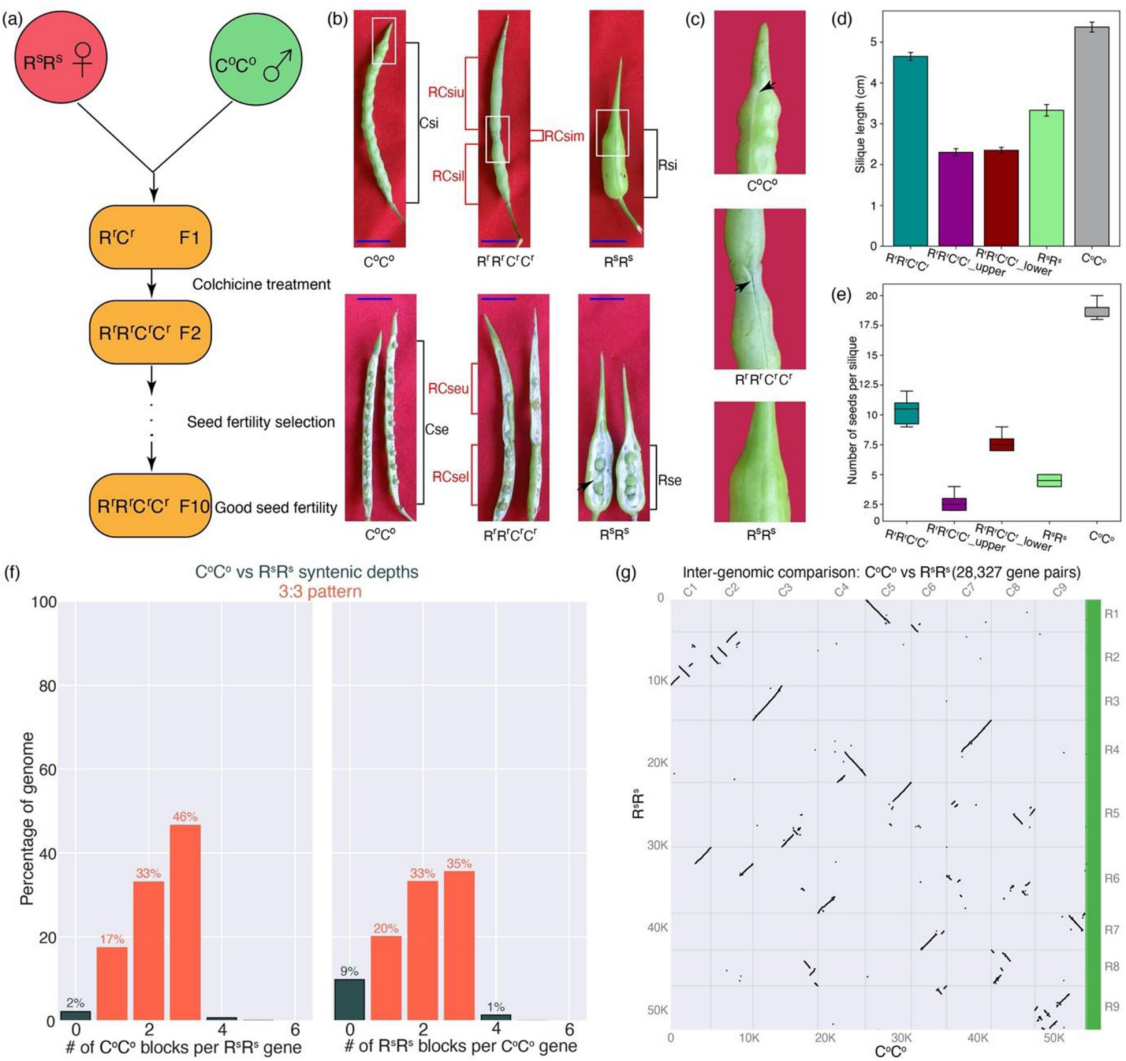
Generation of *Raphanobrassica*, comparison of phenotypes among the hybrid and its diploid progenitors and synteny analysis between R^s^R^s^ and C°C° genomes. **a** The schematic diagram for the generation of allotetraploid *Raphanobrassica*. **b** Comparisons of the whole silique, pod, and seed phenotypes among the hybrid and its diploid parents at 35 DAF. The black arrow indicates a sponge-like membrane enclosing R^s^R^s^ seeds. Scale bars, 1 cm. **c** Magnified images of those from (**b**) in white rectangular boxes. Arrows indicate the dehiscence zones of Csi and RCsil. **d** Silique length (without the tip) of R^s^R^s^, C°C°, R^r^R^r^C^r^C^r^, upper and lower parts of the R^r^R^r^C^r^C^r^ silique at 35 DAF. Error bars, standard deviation. **e**The number of seeds per silique of R^s^R^s^, C°C°, R^r^R^r^C^r^C^r^, upper and lower parts of R^r^R^r^C^r^C^r^ silique. **f** A 3:3 synteny pattern between R^s^R^s^ and C°C° genomes. **g** Dot plot of pairwise synteny between R^s^R^s^ and C°C° genomes. R1-R9 indicated the nine longest scaffolds of the R^s^R^s^ genome. C1-C9 indicated the nine chromosomes of C°C^o^ genome

The intergeneric allotetraploid *Raphanobrassica* (R^r^R^r^C^r^C^r^) was generated by crossing diploid *R. sativus* (R^s^R^s^, maternal parent) and diploid *B. oleracea* var. *alboglabra* (C°C°, paternal parent) following fertility selection for ten generations (Fig. 1a). For convenience, we use the species names and their representative genomes interchangeably in the following text, i.e., R^s^R^s^ for *R. sativus*, C°C° for *B. oleracea,* and R^r^R^r^C^r^C^r^ for *Raphanobrassica*. Siliques of the hybrid exhibit intermediate phenotypes compared to those of the diploid progenitors (Fig. 1b–e and Fig. S1). Specifically, the silique of the hybrid is obviously split into two parts joined by the valve shoulder (RCsim). The upper section of pods (RCsiu) displays more similarities to those of *R. sativus*, and neither of them possesses the seed-bearing septum (Fig. 1b, c). Instead, seeds in RCsiu and Rsi are enclosed by sponge-like membranes. In contrast, RCsil shares more similarities with the silique of C°C° (Csi), and both harbor a seed-bearing septum (Fig. 1b, c). However, the whole silique length of R^r^R^r^C^r^C^r^ (4.65 ± 0.10 cm) is between those of the progenitor R^s^R^s^ (3.33 ± 0.14 cm) and C°C° (5.37 ± 0.12 cm), but it is much more similar to that of C°C°. A similar phenomenon was found in the phenotype of silique width, with RCsiu (4.83 ± 0.17 mm) and RCsil (4.67 ± 0.21 mm) sharing much more similarities with C°C° (3.83 ± 0.17 mm) than R^s^R^s^ (10.00 ± 0.26 mm) (Fig. 1d and Fig. S1). As expected, the number of seeds per silique of R^r^R^r^C^r^C^r^ (10.33 ± 1.21) was between those of R^s^R^s^ (4.17 ± 1.17) and C°C° (18.67 ± 1.03), with more seeds in RCsil (7.67 ± 0.82) than in RCsiu (2.67 ± 0.82) (Fig. 1e). In addition, the color of mature seeds, seed area, and thousand seed weight (TSW) of the hybrid were also between those of the two parents but were much more similar to those of C°C° (Fig. S1). Of note, few differences in the phenotypes of seed area, TSW, and color of mature seeds between RCseu and RCsel were found.

In brief, silique phenotypes of silique structure, silique length, silique width, seed area, the color of mature seeds, and TSW of the hybrid are between those of its parents but very similar to those of C°C°. Conclusively, phenotypes of the whole silique of *Raphanobrassica* are much more similar to those of *B. oleracea* than those of *R. sativus*, even though the developing seeds of the hybrid also share slight similarities with those of *R. sativus* (Fig. 1b), suggesting that global gene expression pattern of *Raphanobrassica* silique is much more similar to its paternal parent than that of its maternal parent.

### Global mRNA transcriptome analysis of different sections of silique

To investigate the gene expression patterns behind these interesting phenomena, we first performed synteny analysis between the paternal (C°C°, assembly BOL) and maternal (R^s^R^s^, assembly Rs1.0) genomes. It was revealed that the synteny depth between the paternal and maternal genomes is a 3:3 pattern (Fig. 1f), and the genome-wide synteny between these two genomes is of high level (Fig. 1g), indicating that many genes of the two genomes are highly homologous. Subsequently, to unravel the global gene expression patterns, RNA-seq of RCsiu, RCsim, RCsil, RCseu, RCsel, Rsi, Rse, Csi, and Cse was performed. All of the generated sequencing reads were processed and mapped to the R^s^R^s^ genome (assembly Rs1.0), C°C° genome (assembly BOL), and integrated genomes of R^s^R^s^ and C°C° (Table S1). Except for Csi3, we obtained approximately 1.4 × 10^9^ clean reads (5.4 × 10^7^ on average), approximately 1.2 × 10^9^ of which (83.4%) were mapped to the corresponding genomes. Most of the mapped reads were uniquely mapped reads (94.2%), and the vast majority of them were mapped to exons (Table S1). Pearson correlation coefficients (PCCs) between these samples ranged from 0.801–0.996, which indicates the high-quality of the biological replicates (Fig. S2). Twelve genes were randomly selected for further RT–qPCR analysis, and the results showed the same expression patterns as the mRNA-seq data (Fig. S3).

**Fig. 2:**
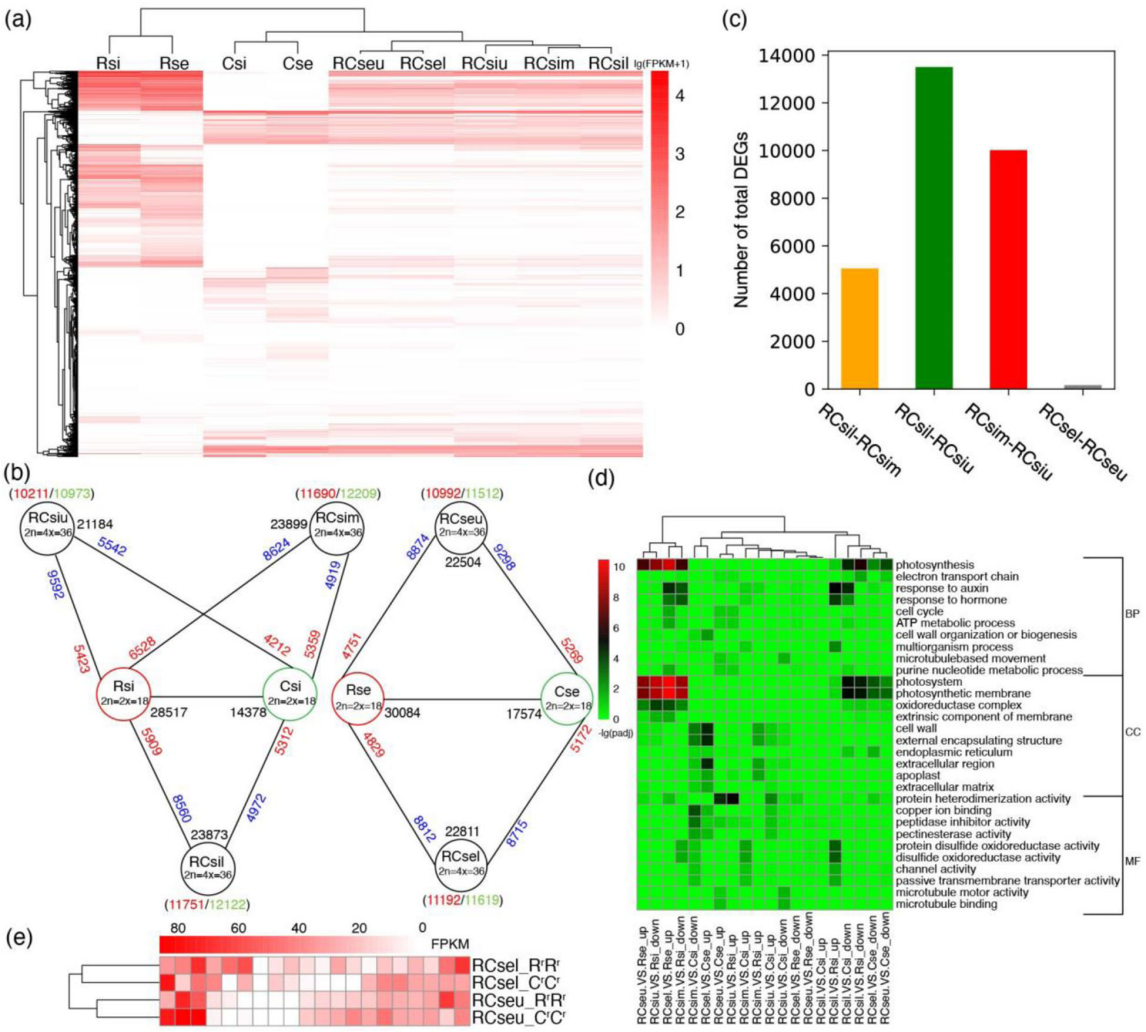
Global transcriptome analysis. **a** Heat map showing the expression levels of 20,000 randomly selected genes from Rsi, Rse, Csi, Cse, RCsiu, RCsim, RCsil, RCseu, and RCsel. **b** Numbers of expressed genes and DEGs of the nine tissues. Total numbers of expressed genes of different tissues are indicated by the black numbers, and the blue and red numbers along the solid black lines reveal the down- and upregulated genes in the five tissues of R^r^R^r^C^r^C^r^ relative to those in the progenitors, respectively. In addition, the red and green numbers in the brackets indicate the genes coming from the R^r^R^r^ and C^r^C^r^ subgenomes, respectively. **c** Total DEGs identified in pairwise comparison among the three sections of pods and between the seeds in the two sections of siliques of the hybrid. **d** Functional categorization of DEGs in GO terms among the nine tissues. **e** Expression levels of 20 randomly selected photosynthesis-related DEGs in R^r^R^r^C^r^C^r^ relative to the diploid progenitors. R^r^R^r^ and C^r^C^r^ indicated the two subgenomes of the hybrid

There were indeed some differences in global gene expression patterns among RCsiu, RCsim, and RCsil, but few were found between those of RCseu and RCsel (Fig. 2a–c). Thus, for convenience, only the transcriptome of RCseu was used for further homoeolog expression bias and expression level dominance analyses in the following text. Moreover, the results also revealed that the global gene expression level distribution of R^r^R^r^C^r^C^r^ was more similar to that of C°C° than that of R^s^R^s^ in all five silique sections (Fig. 2a and Fig. S4).

In total, we identified 59,018 (41,140 known genes and 17,878 novel gene loci) genes as expressed (empirical cutoff value: FPKM ≥1) in at least one of twenty-six samples (except for Csi3). Specifically, a total of 28,517, 14,378, 30,084, 17,574, 21,184, 23,899, 23,873, 22,504 and 22,811 expressed genes were identified in Rsi, Csi, Rse, Cse, RCsiu, RCsim, RCsil, RCseu and RCsel, respectively (Fig. 2b). The three sections of pods and the seeds in the two sections of R^r^R^r^C^r^C^r^ siliques have intermediate numbers of expressed genes compared to those of the two progenitors, which is consistent with the intermediate phenotypes of silique of the hybrid (Fig. 2b). Dramatically, most of the differentially expressed genes (DEGs) in RCsiu, RCseu, and RCsel were downregulated relative to those in the diploid progenitors. However, there were more upregulated DEGs than downregulated DEGs in RCsim and RCsil when compared to Csi (Fig. 2b). Different sets of DEGs were also classified by GO terms. Notably, the upregulated and downregulated DEGs in seeds of R^r^R^r^C^r^C^r^ relative to the R^s^R^s^ genome and the C°C° genome, respectively, were enriched for photosynthesis-related GO terms (Fig. 2d). However, the expression levels of the 20 randomly selected photosynthesis-related DEGs in *Raphanobrassica* were comparable between the subgenomes of R^r^Rr and C^r^C^r^ (Fig. 2e).

Together, the sharp alteration in the global gene expression patterns of pods and seeds of R^r^R^r^C^r^C^r^ relative to those of the parental genomes indicates a “transcriptome shock” in the hybrid genome. The intermediate numbers of expressed genes of pods and seeds of the hybrid between those of the progenitors are also consistent with the intermediate phenotypes of pods and seeds of the hybrid. However, the genomic regulation mechanisms underlying these phenomena remain unclear.

### Analysis of expression profile differences between the upper and lower sections of silique of R^r^R^r^C^r^C^r^

**Fig. 3:**
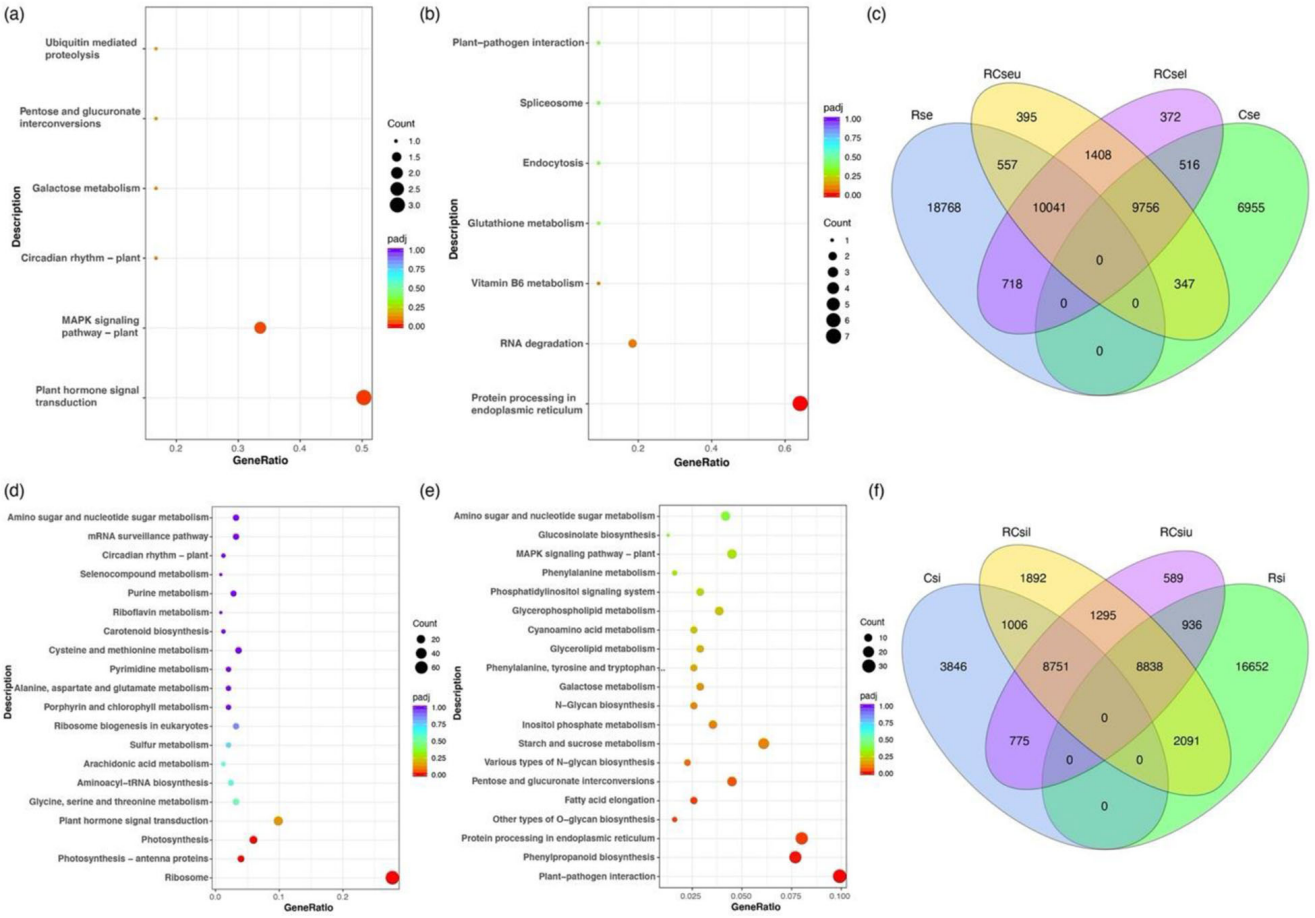
Analysis of expression profile differences between the upper and lower sections of silique of R^r^R^r^C^r^C^r^. **a** KEGG enrichment analysis of upregulated DEGs of RCseu relative to those of RCsel. **b** KEGG enrichment analysis of downregulated DEGs of RCseu relative to those of RCsel. **c** Venn diagram analysis of expressed genes in Rse, Cse, RCseu, and RCsel. **d** KEGG terms of upregulated DEGs of RCsiu relative to RCsil. **e** KEGG terms of downregulated DEGs of RCsiu relative to RCsil. **f** Venn diagram analysis of expressed genes in Rsi, Csi, RCsiu, and RCsil

Although there are few differences in the expression profile between RCseu and RCsel, we want to know what the specific differences are, as well as those between RCsiu and RCsil. KEGG terms of upregulated DEGs of RCseu relative to RCsel were significantly enriched for ‘plant hormone signal transduction’ and ‘MAPK signaling pathway-plant’ (Fig. 3a). Detailed analysis revealed that two of these DEGs (three unique genes in total) negatively regulate ABA signaling, and one positively regulates cytokinin signaling. Suppression of *AHG3* (gene ID: 106295669), a negative regulator of ABA signaling, could significantly accelerate fruit ripening in tomato^34^. However, KEGG analysis of downregulated DEGs of RCseu relative to RCsel was significantly enriched for ‘Protein processing in endoplasmic reticulum’ (Fig. 3b). Interestingly, seven homoeolog gene pairs were detected within the downregulated DEGs of RCseu relative to RCsel, and three pairs (gene ID: 108812972–106312198, 108817415–106299879, and 108857941–106295243) were identified as molecular chaperones that play critical roles in ER stress^35^. A previous study revealed that these molecular chaperones might be required for desiccation tolerance in *Arabidopsis*^36^. These results indicate that the ripening of RCseu might lag behind that of RCsel, which is in line with the phenotype in which the color of RCsel is deeper than that of RCseu (Fig. 1b). Furthermore, Venn diagram analysis revealed a high proportion of coexpressed genes between RCseu and RCsel (20,845, 95.6% in RCseu and 95.9% in RCsel), and the two tissues shared more coexpressed genes with Rse than Cse (Fig. 3c). In addition, the difference in the expression level of specifically expressed genes in RCseu and RCsel was minimal, and the top eight highly specifically expressed genes in RCsel were identified as novel gene loci (Fig. S5). Together, these results indicate that there are subtle, but nearly no, differences in expression profiles between RCseu and RCsel, which is in line with the phenotypes.

In contrast, the percentages of coexpressed genes between RCsil (18,884, 86.7% of total expressed genes) and RCsiu (18,884, 92.5% of total expressed genes) were smaller than those of RCseu and RCsel, and RCsil conferred more specific expressed genes than RCsiu (Fig. 3f). In addition, upregulated and downregulated DEGs of RCsiu relative to RCsil were enriched for different KEGG terms, for example, ‘Ribosome’ and ‘Plant pathogen interaction’, respectively (Fig. 3d, e). Intriguingly, we noticed that the pod wall of RCsiu is thicker but softer than that of RCsil. The GO term of upregulated DEGs of RCsil relative to those of RCsiu was significantly enriched for “cell wall”, thus indicating that RCsil is much more lignified than RCsiu (Fig. S6). We next identified 14 upregulated homoeologous gene pairs in RCsil enriched for the “cell wall”, which might explain the more rigid pod wall of RCsil than that of RCsiu (Fig. S6). Furthermore, pod shattering was clearly observed in RCsil, but not RCsiu, during harvest. A previous study showed that the *IND* gene is required for pod shattering^37^ and that the *RPL* gene could prevent replum cells from developing into valve margins^38^. Analysis of the expression level of the two aforementioned genes revealed that *RPL* is significantly expressed in Rsi, and the expression level in RCsiu is higher than that of RCsil. However, *IND* is mainly expressed in Csi and RCsil, which might explain the different phenotypes in pod shattering between RCsiu and RCsil (Fig. S6). Nevertheless, more transcriptome data in the early developmental stages of siliques are required to study these differences. Together, these results elucidate the expression profile differences between the upper and lower sections of siliques of the hybrid.

### Genome-wide unbalanced biased expression toward CC in pods and seeds of R^r^R^r^C^r^C^r^

**Fig. 4:**
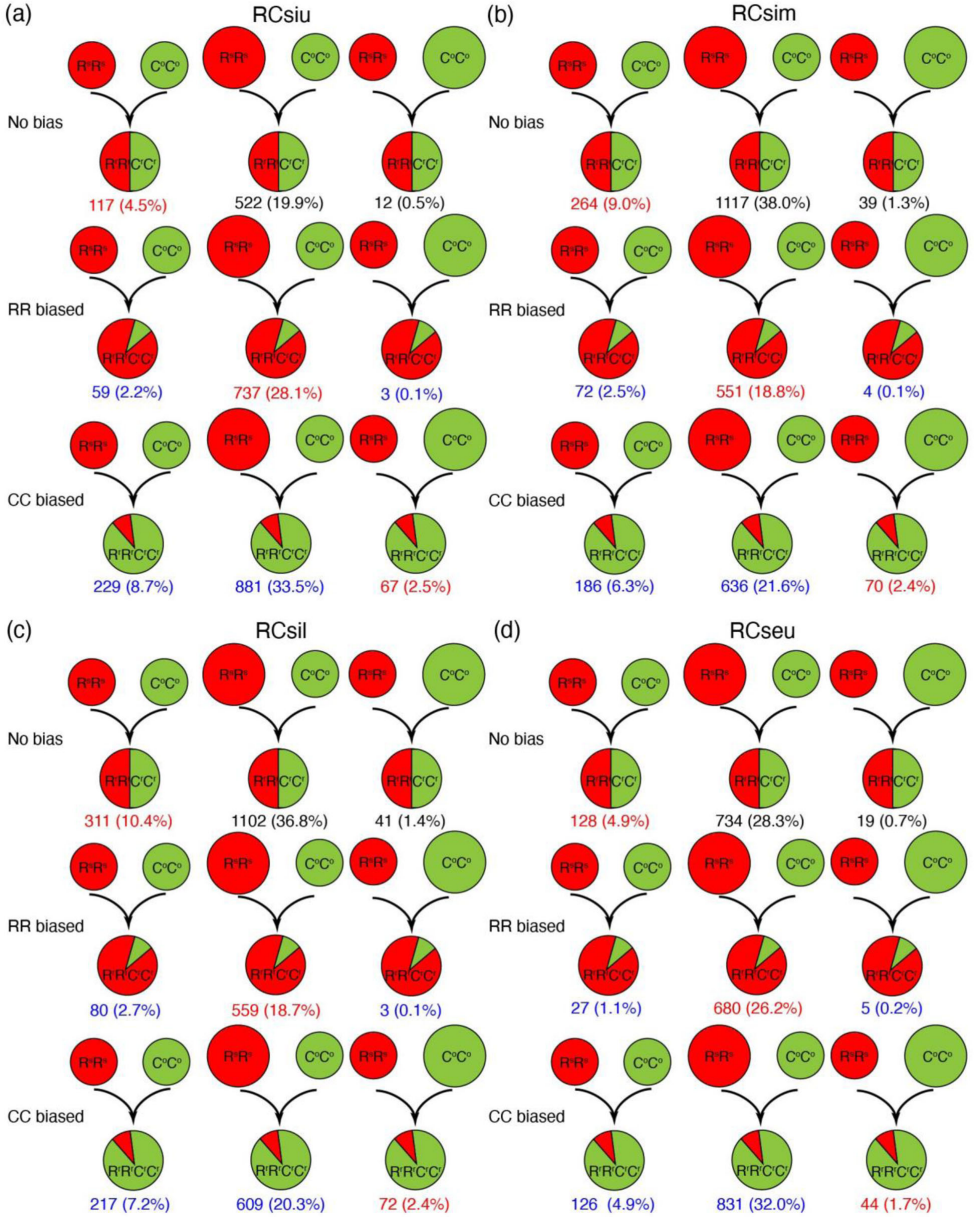
Analysis of homoeolog expression bias of homoeologous gene pairs in R^r^R^r^C^r^C^r^. **a** Homoeolog expression bias analyses of RCsiu, (**b**) RCsim, (**c**) RCsil and (**d**) RCseu. The numbers of homoeolog pairs and their proportions to the total numbers of analyzed pairs are listed. The sizes of the circles in the diploid progenitor R^s^R^s^ and C^o^C^o^ indicated the relative expression levels of the orthologs (homoeologs in the hybrid), while relative expression levels of the homoeologs in R^r^R^r^C^r^C^r^ were indicated by the area ratio of the circles. In addition, the numbers in red indicate that the expression patterns of homoeologs in R^r^R^r^C^r^C^r^ maintained parental conditions, while those in blue indicate novel expression bias in the hybrid

Homoeolog expression bias refers to the preferential expression of one homoeolog relative to the other in the hybrids^22^. To study the homoeolog expression bias in RCsiu, RCsim, RCsil, and RCseu, the genes that were expressed in at least one progenitor and where both homoeologs were expressed in *Raphanobrassica* were analyzed. First, the BBH method was adopted to identify 23,566 orthologous gene pairs between R^s^R^s^ and C°C° genomes (homoeologous gene pairs of the hybrid subgenomes). Then, a total of 2627, 2939, 2994, and 2594 homoeologous gene pairs were used for expression bias analysis in RCsiu, RCsim, RCsil, and RCseu, respectively. Unbalanced biased expression with a preference toward CC was observed in all four tissues, with a total of 1177 (44.7%), 892 (30.3%), 898 (29.9%), and 1001 (38.6%) homoeologous gene pairs detected in RCsiu, RCsim, RCsil, and RCseu, respectively (Fig. 4). In contrast, the numbers (percentages) of homoeologous gene pairs with RR-biased expression were 799 (30.4%), 627 (21.4%), 642 (21.5%), and 712 (27.5%) in RCsiu, RCsim, RCsil, and RCseu, respectively, which were lower than those of the CC-biased pairs (Fig. 4).

Remarkably, more gene pairs showed RR-biased expression in RCsiu and RCseu than in RCsim and RCsil, which might explain why the phenotypes of RCsiu and RCseu share more similarities to *R. sativus* than those of RCsim and RCsil (Fig. 4, Fig. 1b–d and Fig. S1). In addition, a total of 651 (24.9%), 1420 (48.3%), 1454 (48.6%), and 881 (33.9%) gene pairs showed no bias in RCsiu, RCsim, RCsil, and RCseu of *Raphanobrassica*, respectively (Fig. 4). Impressively, many homoeologous gene pairs that displayed novel bias were observed in RCsiu (1 172, 44.5%), RCseu (989, 38.2%), RCsim (898, 30.5%), and RCsil (909, 30.3%), among which most of the homoeolog pairs with preexisting biased expression toward RR reverted to biased expression toward CC (Fig. 4). Furthermore, approximately one-third of the respective homoeolog pairs analyzed were maintained under progenitor conditions (Fig. 4). Collectively, the analysis of homoeolog expression bias revealed unbalanced biased expression with a preference toward CC, and more homoeolog gene pairs showed RR-biased expression in RCsiu and RCseu than in RCsim and RCsil. The much more homoeolog gene pairs that show RR-biased expression might explain why the phenotypes of RCsiu and RCseu share more similarities to *R. sativus* than those of RCsim and RCsil.

### Genome-wide expression level dominance biased toward CC in pods and seeds of *Raphanobrassica*

**Fig. 5:**
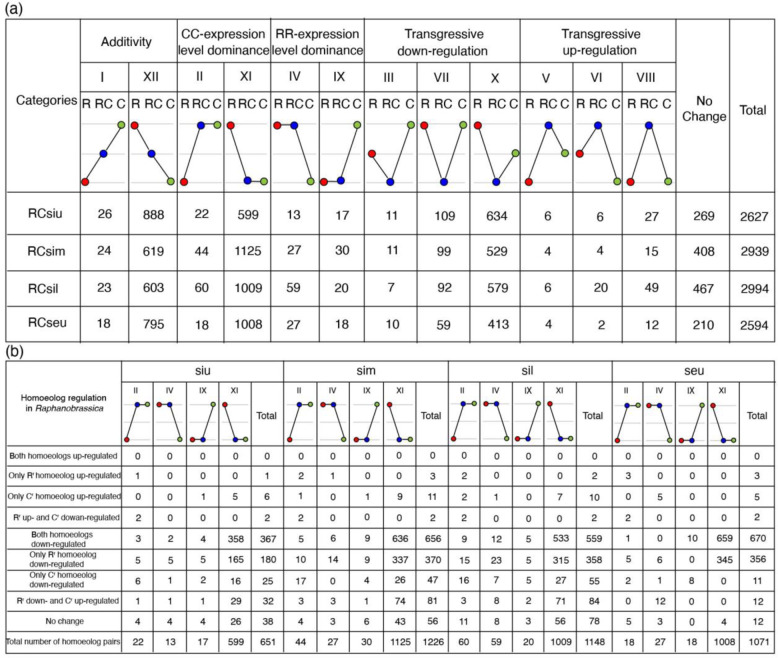
Expression level dominance and relationships between the ELD and individual homoeolog expression levels. **a** ELD in the three sections of pods and RCseu of *Raphanobrassica*, reflecting the collective expression of homoeolog pairs compared to those in the progenitors. **b** Relationships between ELD and individual homoeolog expression levels explained the phenomenon of ELD in the four tissues

Expression level dominance (ELD) is another important concept distinct from homoeolog expression bias, which refers to the total expression level of a homoeolog pair in allopolyploids compared to its two progenitors^22^. To explore the ELD in RCsiu, RCsim, RCsil, and RCseu of *Raphanobrassica*, we only focused on the genes that are expressed in at least one progenitor and where both homoeologs are expressed in *Raphanobrassica*, and these gene pairs were classified into 12 categories by comparing the total expression of the homoeolog pairs in R^r^R^r^C^r^C^r^ to those in the progenitors (Fig. 5a).

The results revealed that the numbers of homoeolog pairs displaying CC expression level dominance (ELD-CC, categories II and XI) were much larger than those displaying RR expression level dominance (ELD-RR, categories IV and IX) in RCsiu (621/23.6% vs. 30/1.1%), RCsim (1 169/39.8% vs. 57/1.9%), RCsil (1069/35.7% vs. 79/2.6%), and RCseu (1026/39.6% vs. 45/1.7%) (Fig. 5a). Notably, the homoeolog pairs that display ELD-CC are mainly from category XI, indicating the significant downregulation of R^r^R^r^ homoeologs compared to those in *R. sativus*. Additionally, 914 (34.8%), 643 (21.9%), 626 (20.9%) and 813 (31.3%) homoeologous gene pairs showed additivity in RCsiu, RCsim, RCsil and RCseu, respectively (Fig. 5a). Furthermore, a total of 754 (28.7%), 639 (21.7%), 678 (22.6%), and 482 (18.6%) homoeolog pairs displayed transgressive downregulation (categories III, VII, and X) (Fig. 5a). However, only a total of 269 (10.2%), 408 (13.9%), 467 (15.6%), and 210 (8.1%) homoeolog pairs exhibited equivalent expression (“No change” in Fig. 5a) in RCsiu, RCsim, RCsil, and RCseu, respectively. Moreover, the functional categorizations of different sets of homoeolog pairs showing ELD were also studied in GO terms, with three sets of genes (siu-C-ELD, sil-C-ELD, and seu-C-ELD) enriched for “protein heterodimerization activity” and “rRNA binding” (Table S2). However, genes of sim-C-ELD were enriched for photosynthesis (Table S2).

The relationship between ELD and individual homoeolog expression levels in the RCsiu, RCsim, RCsil, and RCseu of *Raphanobrassica* was also investigated by comparing the individual homoeolog expression levels to those in the diploid progenitors. As expected, the main reason for ELD is that numerous homoeologs or R^r^R^r^ homoeologs were downregulated compared to those in the diploid progenitors, with 547 (367 + 180, 84.0%), 1 026 (656 + 370, 83.7%), 917 (559 + 358, 79.9%) and 1 026 (670 + 356, 95.8%) homoeolog pairs detected in RCsiu, RCsim, RCsil, and RCseu, respectively (Fig. 5b). Together, these results reveal that the expression level dominance in RCsiu, RCsim, RCsil, and RCseu is toward CC, and the main reason for ELD is due to the downregulated expression of both R^r^R^r^ and C^r^C^r^ homoeologs or the downregulated expression of R^r^R^r^ homoeologs.

## Discussion

Hybridization between different species promotes genome evolution and the formation of new crops by genetic recombination, which is usually used to generate novel cultivars with novel genetic traits. Hybrids usually display hybrid vigor (heterosis), disease resistance, and some other traits in future generations^12^. For example, the Chinese cabbage F_1_ hybrid Xin No. 3, a popular leafy crop species generated from hybridization between the inbred lines SD and JEY, displays strong heterosis for biomass during vegetative growth and development^4^. In this study, the intergeneric hybrid derived from the hybridization of *R. sativus* (RR, 2*n* = 18) and *B. oleracea* (CC, 2*n* = 18) was used for the analysis of mRNA expression profiles. GISH experiments showed that *Raphanobrassica* has the expected chromosome number through many generations of fertility selection^31,32^. *R. sativus* was reported to resist different diseases^39^. The transfer of the R^s^R^s^ genome from *R. sativus* into *Raphanobrassica* may provide an important resource for disease resistance traits. Our previous study showed the resistance of *Raphanobrassica* to powdery mildew disease, which may provide an important bridge for disease resistance breeding^40^. Moreover, plenty of evidence has shown that *Raphanobrassica* has resistance to clubroot disease, beet cyst nematodes, and effective crossability with *Brassica* species^33^. Therefore, *Raphanobrassica* generated in this work provides a potential new genetic resource for practical use in breeding clubroot-resistant cultivars of *Brassica* crops. In addition, *Raphanobrassica* is superior to its two progenitors in dry weight and crude protein content^32^, which would serve as a useful fodder crop, and the dehiscence characteristic of RCsil makes the siliques easily harvested mechanically. However, how the interaction between the R^s^R^s^ genome of *R. sativus* and the C°C° genome of *B. oleracea* affects chromosome rearrangement in *Raphanobrassica* remains unclear. Nevertheless, our global gene expression pattern analysis reported here will help the research community broadly understand the consequence of the incorporation of R^s^R^s^ and C°C° genomes in the silique of *Raphanobrassica*.

Transcriptome shock in the hybrid refers to extensive changes to patterns of parental gene expression^41^. In this study, transcriptome shock was observed in all five tissues of R^r^R^r^C^r^C^r^. A previous study revealed that transcriptome shock might be caused by interspecific hybridization and subsequently ameliorated by genome duplication^42^. Thus, we speculated that changes in the expression patterns of the diploid F1 hybrid might be more extensive than those of the allotetraploid hybrid when compared to the diploid progenitors. In addition, altered chromatin compaction and histone methylation in the hybrid^43^, as well as cis- and trans-regulatory divergence between progenitor species^44^, may also explain the transcriptome shock observed here. Considering that most DEGs in R^r^R^r^C^r^C^r^ are downregulated relative to those of the maternal parent *R. sativus*, we speculated that chromatin of the R^r^R^r^ subgenome is much more compact than that of the C^r^C^r^ subgenome, and this hypothesis could be confirmed by ATAC-seq and Hi-C in future studies.

Homoeolog expression bias has been broadly studied in the past decade^13,15,45–47^. Interestingly, in our study, the silique of *Raphanobrassica* was split into two parts, and homoeolog expression bias showed a preference for CC. However, more homoeolog gene pairs showed RR-biased expression in RCsiu and RCseu than in RCsim and RCsil. Strikingly, homoeolog expression and induction bias were revealed in *Triticum aestivum* when bread wheat was infected by the fungal pathogen *Fusarium pseudograminearum*^45^. Once infected by the aforementioned fungal pathogen, B and D homoeologs exhibit stronger responses to the infection than A homoeologs, which implies distinctions of homoeolog expression bias under different biotic and abiotic stresses in many resynthesized or natural hybrids. Indeed, some recent studies have also revealed that the expression levels of certain homoeologous genes are altered under heat tolerance, water stress, and iron deficiency in polyploid wheat^48–50^. Hence, homoeolog expression bias of pods and seeds of *Raphanobrassica* under different stresses still remains to be studied.

ELD has also been widely explored in many resynthesized hybrids^13,15,17,51,52^. The parental genome toward which ELD of the synthetic hybrid biases is consistent with the similarity of phenotypes between the hybrid and parents. For example, in our study, ELD of pods and seeds of R^r^R^r^C^r^C^r^ showing preference toward CC is consistent with the fact that phenotypes of the whole silique of *Raphanobrassica* are similar to those of *B. oleracea*. Hence, we could infer the ELD of the resynthesized hybrid by the similarity of the phenotypes between the progenitors and the hybrid. In addition, there is different ELD biased expression in different organs within the same organism. For example, one previous study revealed that the gene in immature leaves of *Raphanobrassica* displays genome-wide ELD bias toward RR, which is different from that of the pods and seeds in the present study^51^. Intrigued, the ELD of different developmental stages of pods and seeds of *Raphanobrassica* still remains to be explored to comprehensively understand the relationships of global gene expression patterns between the hybrid and its two diploid progenitors.

Collectively, transcriptome shock was observed in the silique of *Raphanobrassica* due to the incorporation and interaction of the two parental genomes, which might lead to the split of the silique into two parts. The upper and lower sections of siliques of the hybrid exhibit expression profile differences, and the related genes are involved in different biological processes. Many more genes in the silique of *Raphanobrassica* exhibit homoeolog expression bias and expression level dominance toward *B. oleracea* than those toward *R. sativus*. Nonetheless, RCsiu and RCseu have more RR-biased expression genes than RCsim and RCsil, which might explain why the phenotypes of RCsiu and RCseu of the hybrid share more similarities to *R. sativus* than those of RCsim and RCsil.

In conclusion, our mRNA transcriptome profiles are consistent with the observed phenotypes and unravel the tight correlation between the phenotypes and global gene expression patterns in the hybrid and its parents. Our results reported here provide a good reference to study plant polyploidy and mine potential candidate genes responsible for specific phenotypes. However, whether the phenotypes are tightly correlated with other multiomics data in the synthesized hybrid and its parents remains to be explored.

## Materials and methods

### Plant materials and sampling

The diploid maternal *R. sativus* (2*n* = 2*x* = 18) and paternal *B. oleracea* var. *alboglabra* (2*n* = 2*x* = 18) were used as parental lines to generate allotetraploid *Raphanobrassica* (2*n* = 4*x* = 36) by crossing, and the hybrid chromosomes were doubled in the F_1_ generation by colchicine treatment followed by seed fertility selection^32^. All of the plant materials were planted in Wuhan, China. Seeds with good fertility were obtained in the F_10_ generation, and GISH analysis revealed that the hybrid genome contained all 18 chromosomes from R^s^R^s^ and all 18 chromosomes from C°C°^32^.

### Library construction, Illumina sequencing, read mapping and DEG analysis

Whole pods (Rsi and Csi), seeds (Rse and Cse) of siliques of the diploid progenitors, three sections of the whole pods (RCsiu, RCsim, and RCsil) and all seeds in the two sections of the siliques (RCseu and RCsel) of the hybrid, with three biological replicates of each sample, were obtained at 35 DAF for total mRNA isolation and sequencing library construction.

For RNA-seq, RNA library preparation for each sample was performed according to the manuals provided by Illumina. The aforementioned 27 sequencing libraries were sequenced on the Illumina HiSeq 2000 platform. The raw data were filtered by NGS QC Toolkit^53^ to obtain high-quality clean reads. The total clean reads of *R. sativus* and *B. oleracea* obtained after filtration were mapped to the R^s^R^s^ and C°C° genomes, respectively, while those of R^r^R^r^C^r^C^r^ were mapped to the integrated genomes of R^s^R^s^ and C°C° using HISAT software (Table S1)^54^. We also mapped the clean reads of *R. sativus* and *B. oleracea* to the C°C° and R^s^R^s^ genomes to examine whether erroneous mapping existed in the hybrid, and some of the randomly selected results were further confirmed by RT–qPCR. The novel gene loci were predicted by StringTie^55^ and annotated by the Pfam database^56^. The mapping results were also processed by StringTie to obtain FPKM for all 27 samples, and the genes whose FPKM ≥ 1 were identified as expressed. Differentially expressed gene analysis between the 27 samples was conducted using the R package DEseq^57^, and genes that exhibited a difference of at least twofold with a corrected *P* value ≤0.05 were regarded as significantly differentially expressed.

### GO and KEGG enrichment analysis

GO and KEGG enrichment analyses for the DEGs were conducted using the R package clusterProfiler^58^ and the KEGG database^59^, respectively. The GO and KEGG terms of the three ontologies exhibiting *p*_adj_≤0.05 were regarded as significantly enriched.

### Annotation of homoeologous gene pairs in *Raphanobrassica*

For convenience, we considered that most of the homoeologs remained in a one-to-one relationship after polyploidization within the subgenomes of R^r^R^r^C^r^C^r^, although the homoeologous relationships are not necessarily one-to-one^60^. Based on this, we adopted the BBH method to infer homoeologs^61^, although this method has inherent drawbacks^62^. Thus, as shown in Fig. S7, the 43,882 gene models of C°C° and the 48,203 gene models of R^s^R^s^ were blasted against each other using the blastn program^63^ with a search cutoff e-value of 1e^−10^ to identify the orthologous gene pairs, which were termed homoeologous gene pairs in the hybrid. Gene pairs with a sequence identity > =90% were regarded as orthologous gene pairs in the two progenitors or homoeologous gene pairs in the hybrid.

### Analysis of homoeolog expression bias and expression level dominance

In the process of homoeolog expression bias and ELD analyses, we only paid attention to the genes that were expressed in at least one progenitor and where both homoeologs were expressed in *Raphanobrassica*. Student’s *t*-test (*P* < 0.05) was used to compare the expression level of each homoeologous gene pair in the two progenitors (R^s^R^s^ vs. C°C°) and the *Raphanobrassica* hybrid (R^r^R^r^ vs. C^r^C^r^) in the process of homoeolog expression bias according to previously described methods^13^. During the analyses of ELD in different tissues, the collective expression level of a homoeologous gene pair in the *Raphanobrassica* hybrid was compared to that of the diploid progenitors, that is, (R^r^R^r^ + C^r^C^r^) vs. R^s^R^s^ and (R^r^R^r^ + C^r^C^r^) vs. C°C°, using Student’s *t*-test (*P* < 0.05) as described previously^13^. Furthermore, twelve possible bins were classified according to Yoo et al.^52^.

### Collinearity analysis

The protein, CDS, and mRNA FASTA files and GFF files for *B. oleracea* and *R. sativus* were retrieved from the NCBI genome database, and only genes of *R. sativus* from the nine longest scaffolds were used for analysis. Moreover, only the first transcript was used when the gene had more than one transcript. Homologous genes within the self-genome or cross-genome were obtained using BLASTP (blast−2.9.0+)^64^ with a cutoff e-value of 1e^−10^, and only the top five hits were used for downstream collinearity analysis using the *MCSanX* package^65^.

### RT–qPCR validation

The expression level of mRNA was detected in the nine tissues of *R. sativus*, *B. oleracea,* and hybrids using quantitative reverse transcription-PCR (RT–qPCR). For mRNA expression level detection, 1 μg of total RNA was reverse-transcribed using SuperScript III Reverse Transcriptase (Invitrogen) and oligo (dT)18 according to the manufacturer’s protocol. The qPCR experiment was carried out using an ABI 7300 (ABI), and each reaction was performed in triplicate. U6 RNA was set as an internal reference gene for mRNA expression detection. The primers for mRNA RT–qPCR are listed in Table S3.

## Supplementary information

Supporting information

## Data Availability

Sequencing data of *R. sativus*, *B. oleracea,* and the hybrid *Raphanobrassica* were deposited in the SRA Database in NCBI (Accession numbers: SRR12191747, SRR12191748, SRR12191749, SRR12191750, SRR12191751, SRR12191752, SRR12191753, SRR12191754, SRR12191756, SRR12191757, SRR12191758, SRR12191759, SRR12191760, SRR12191761, SRR12191762, SRR12191763, SRR12191764, SRR12191765, SRR12191766, SRR12191767, SRR12191768, SRR12191769, SRR12191770, SRR12191771, SRR12191772, SRR12191773).
