# Supplementary material for: Genome-wide unbalanced expression bias and expression level dominance toward Brassica oleracea in artificially synthesized intergeneric hybrids of Raphanobrassica
Source: Hortic Res. 2021 Dec 1;8:246. doi: 10.1038/s41438-021-00672-2 (PMC8633066; doi:10.1038/s41438-021-00672-2)
Supplement: Supplementary file 1 — Supporting information [file 41438_2021_672_MOESM1_ESM.docx]

**Supporting information**


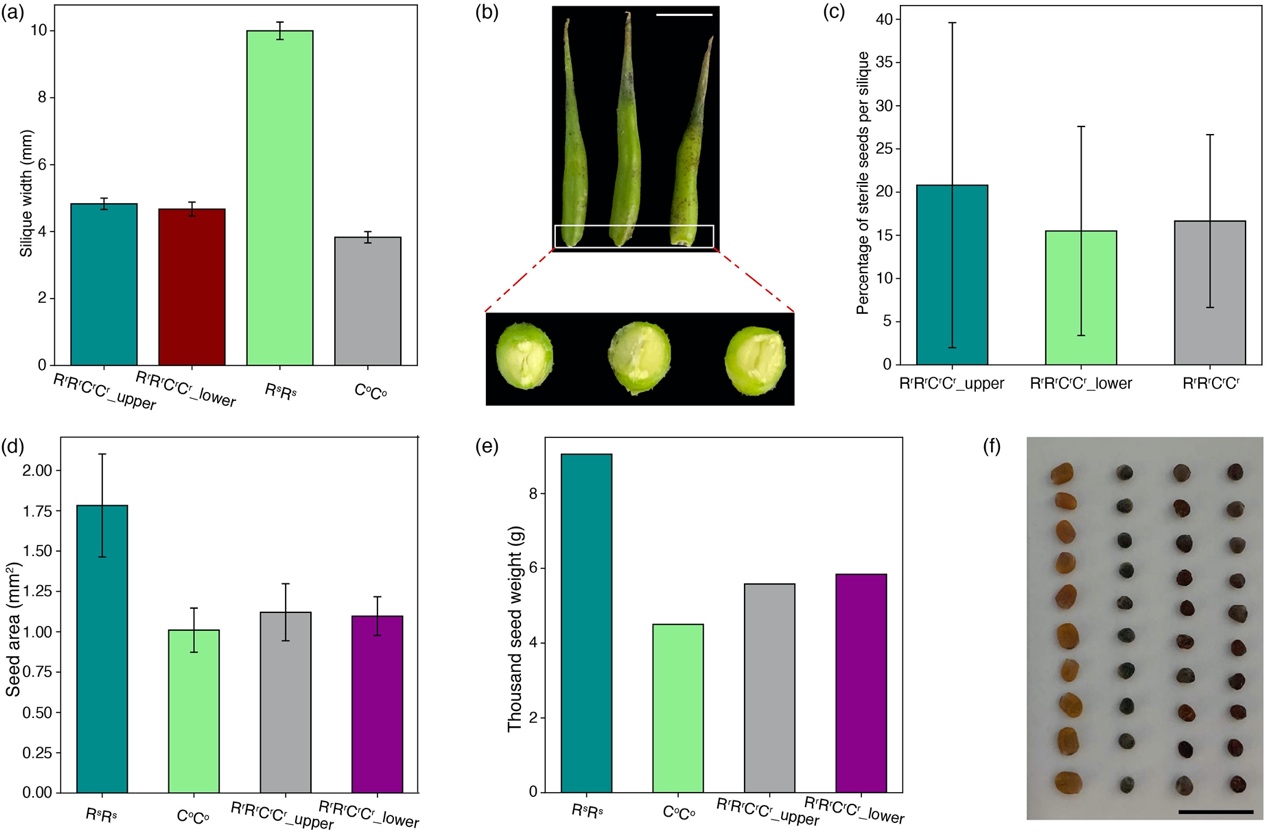


**Fig. S1.** Phenotypic analysis of siliques and seeds of R^s^R^s^, C^o^C^o^ and R^r^R^r^C^r^C^r^. (a) silique width of R^s^R^s^, C^o^C^o^, upper and lower parts of R^r^R^r^C^r^C^r^ silique at 35DAF. Error bars, standard deviation. (b) Crosscutting structure of the valve joint (RCsim) that connects RCsiu and RCsil. Scale bar, 1 cm. (c) Percentage of sterile mature seeds per silique of the hybrid. Error bars, standard deviation. (d) Mature seed area of R^s^R^s^, C^o^C^o^, seeds in upper and lower parts of R^r^R^r^C^r^C^r^ silique. Error bars, standard deviation. (e) Thousand seed weight (mature seeds) of R^s^R^s^, C^o^C^o^, seeds in upper and lower parts of R^r^R^r^C^r^C^r^ silique. (f) Morphology of mature seeds of Rse, Cse, RCseu and RCsel (from left to right). Scale bar, 1cm.


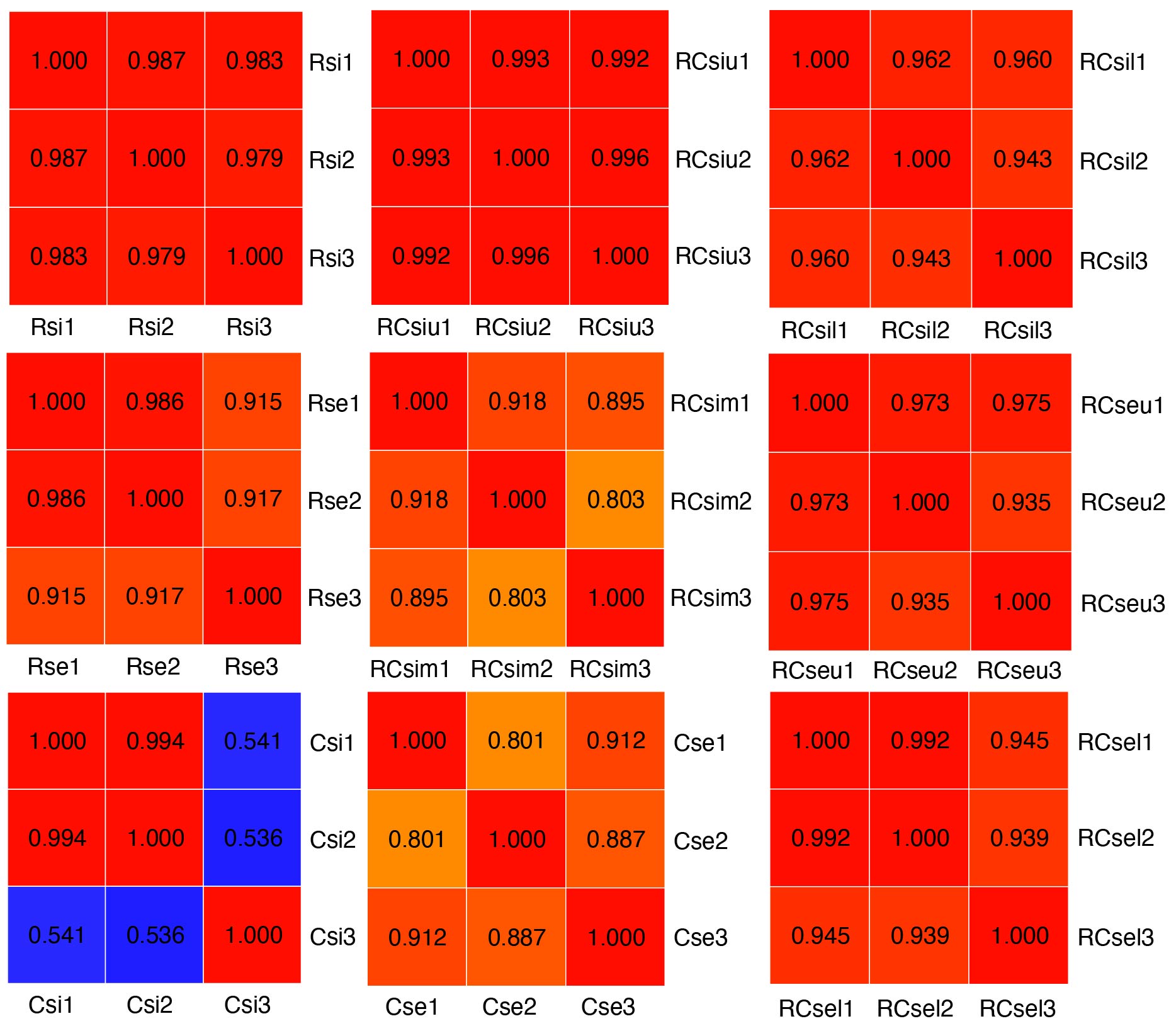


**Figure S2.** PCC analysis among the three biological replicates in the nine tissues.


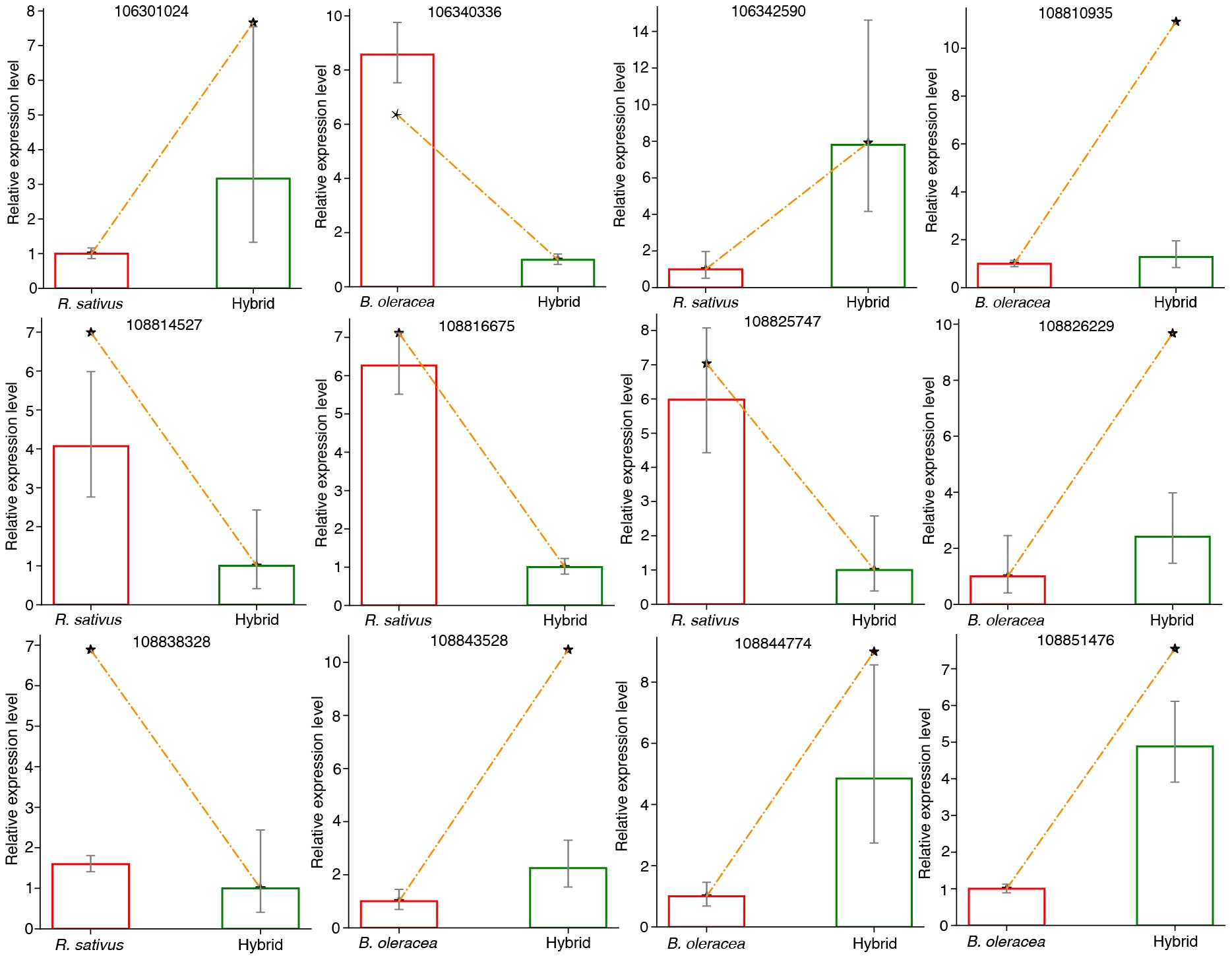


**Figure S3.** RT-qPCR confirmation of mRNA-seq data. Error bars represent the standard deviation from three biological replicates. The relative expression levels in the transcriptome data were indicated by the points connected by dashed lines.


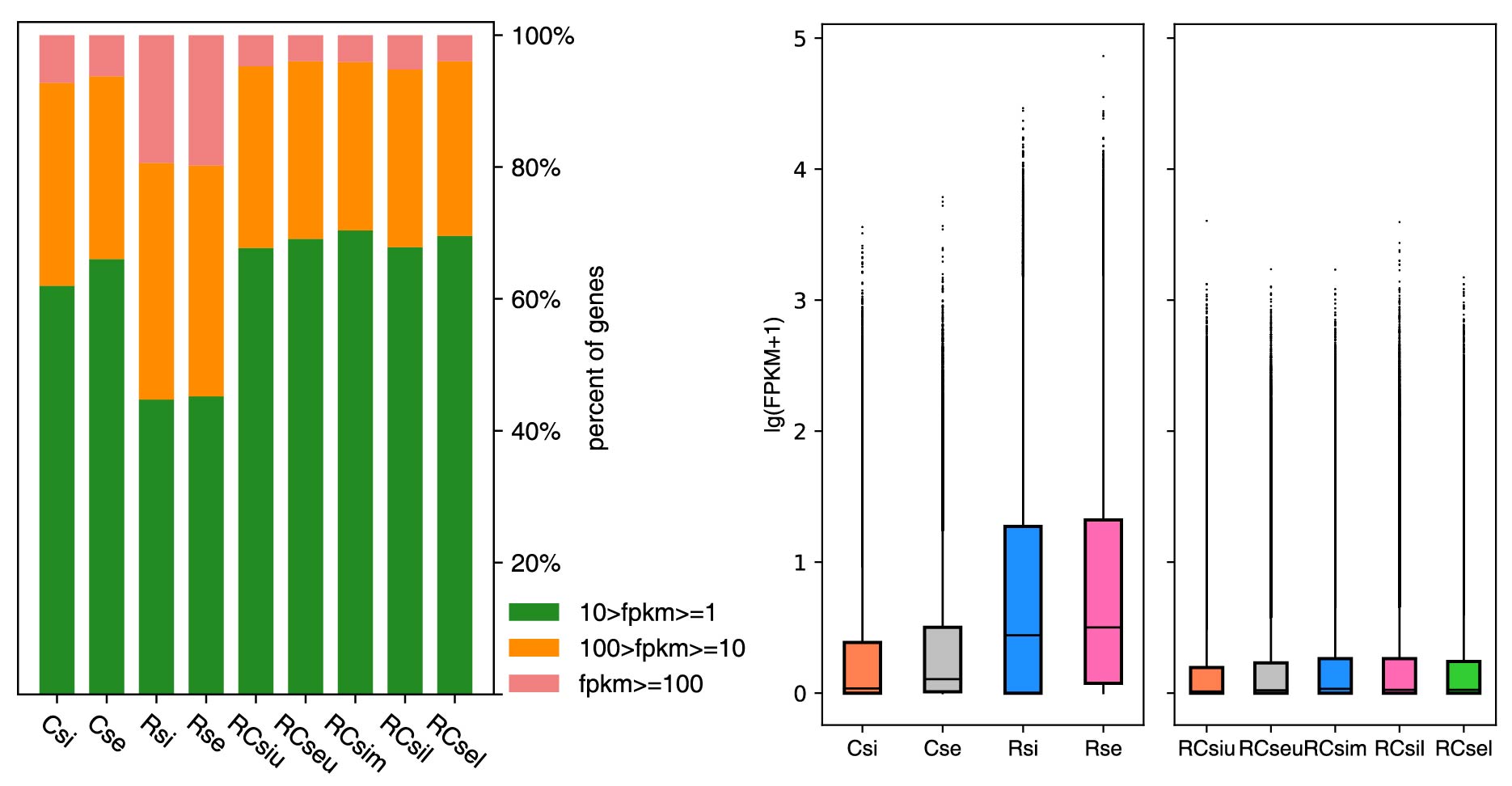


**Figure S4.** Expression level distribution in RCsiu, RCsim, RCsil, RCseu, RCsel, Rsi, Rse, Csi and Cse.


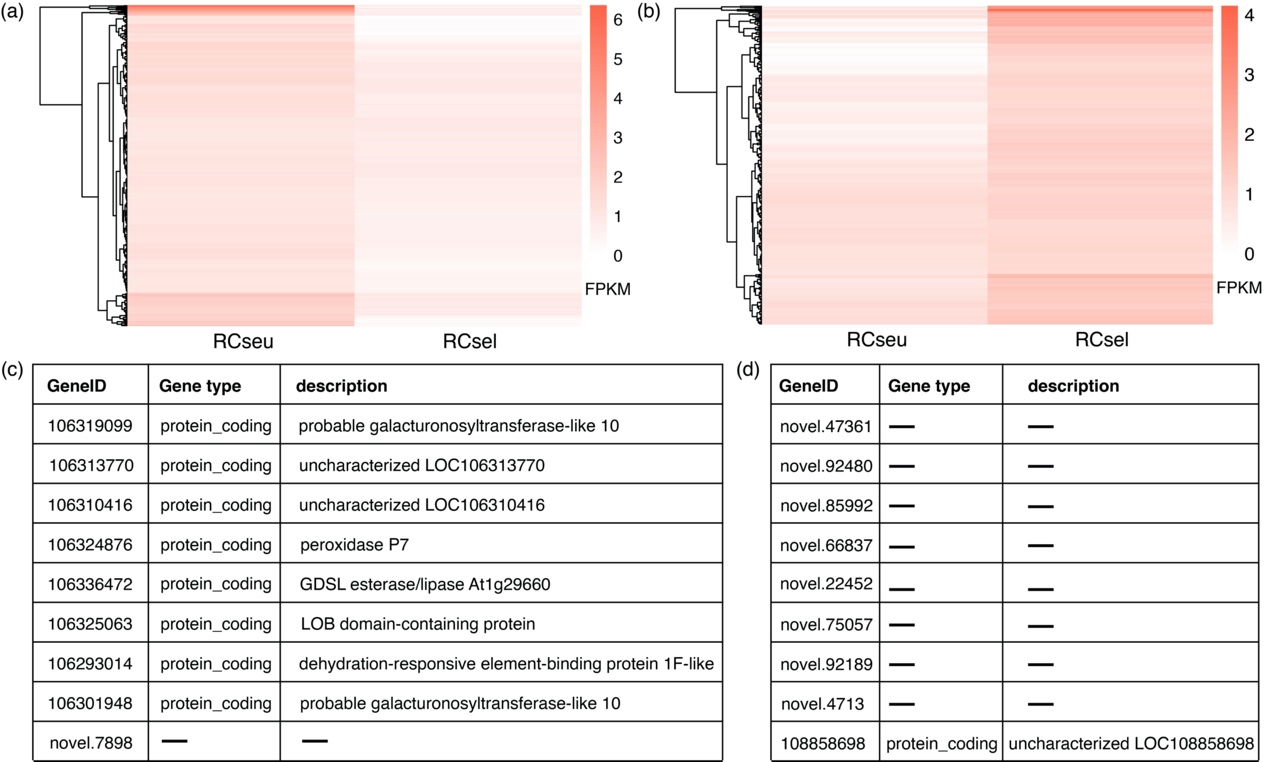


**Figure S5.** Analysis of specifically expressed genes in RCseu and RCsel. (a) Comparison of expression level of RCseu specifically expressed genes between RCseu and RCsel. (b) Comparison of expression level of RCsel specifically expressed genes between RCseu and RCsel. (c) Top nine highly specifically expressed genes in RCseu. (d) Top nine highly specifically expressed genes in RCsel.


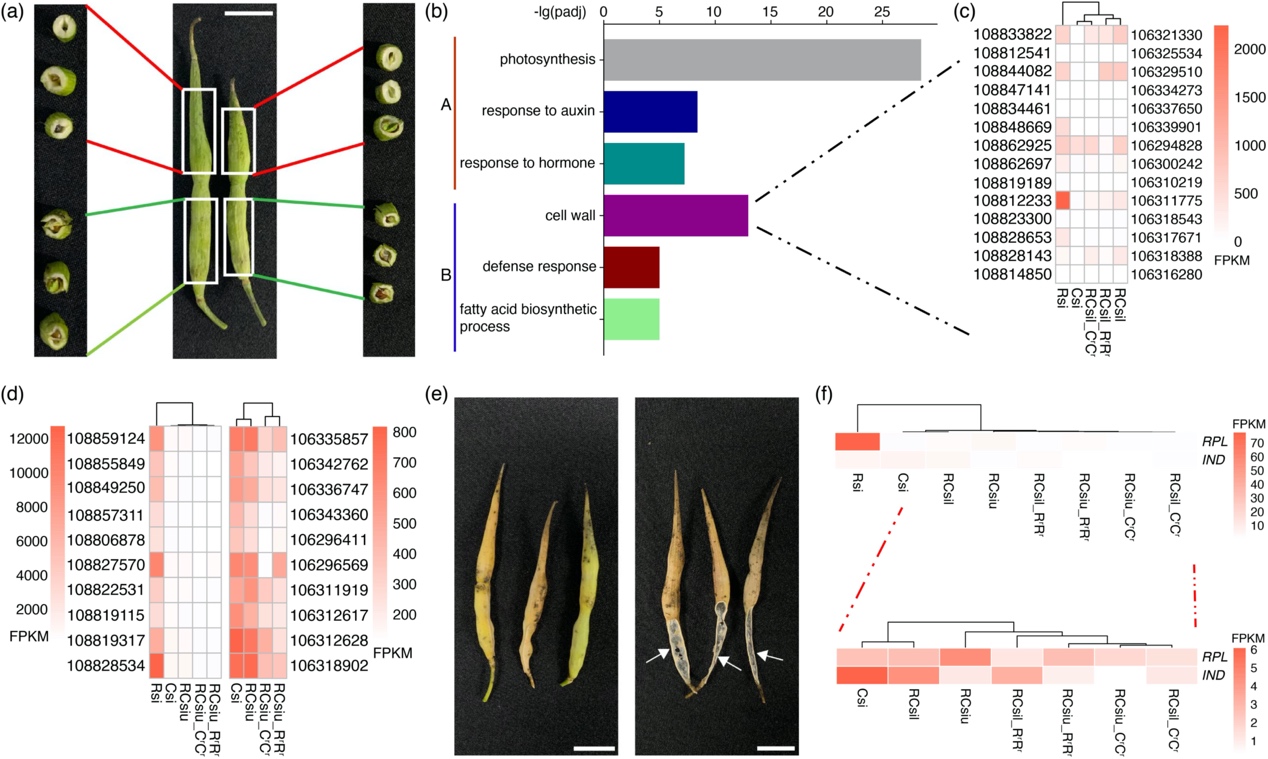


**Figure S6.** Identification of candidate genes responsible for different phenotypes between RCsiu and RCsil. (a) Crosscutting structure of RCsiu and RCsil. Scale bar, 1cm. (b) GO enrichment analysis of DEGs between RCsiu and RCsil. A, GO terms for up-regulated DEGs of RCsiu relative to those of RCsil; B, GO terms for up-regulated DEGs of RCsil relative to those of RCseu. (c) Expression level of homoeologous gene pairs enriched for cell wall in RCsil, Rsi and Csi. (d) Expression level of homoeologous gene pairs enriched for photosynthesis in RCsiu, Rsi and Csi. (e) Pod shattering characteristic in RCsil. White arrows indicated the septum. Scale bar, 1cm. (f) Expression level of *RPL* and *IND* genes in RCsiu, RCsil, Rsi and Csi.


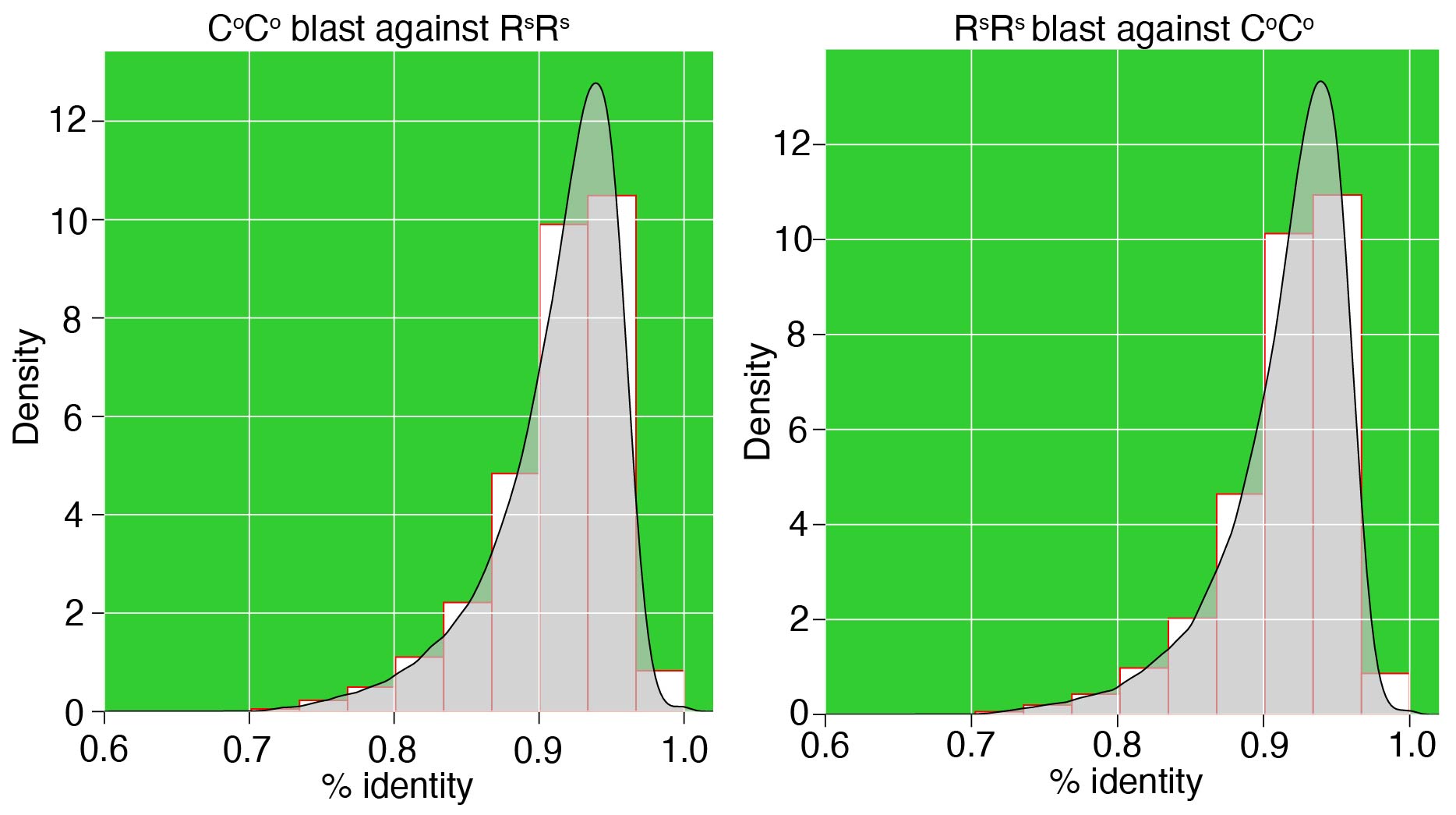


**Figure S7.** Comparative analysis between R^s^R^s^ gene models and C^o^C^o^ gene models using blastn program.

**Table S1.** Summary of read data generated and mapping results

| **Sample^a^** | **Total reads** | **Total mapped reads** | **Uniquely mapped reads** | **Mapping on exons** |
| --- | --- | --- | --- | --- |
| Rsi1 | 56664056 | 45285821(79.92%) | 41167501(72.65%) | 93.84% |
| Rsi2 | 46752904 | 37641441(80.51%) | 34187505(73.12%) | 93.97% |
| Rsi3 | 43267052 | 34248454(79.16%) | 31099297(71.88%) | 93.94% |
| Rse1 | 55087560 | 43079082(78.2%) | 39190100(71.14%) | 94.65% |
| Rse2 | 62798570 | 50439529(80.32%) | 45848655(73.01%) | 94.44% |
| Rse3 | 54646086 | 43823466(80.2%) | 39756098(72.75%) | 93.57% |
| Csi1 | 54732530 | 47798432(87.33%) | 46649059(85.23%) | 94.55% |
| Csi2 | 58480996 | 51170313(87.5%) | 49937814(85.39%) | 94.52% |
| Cse1 | 43251490 | 38057551(87.99%) | 36974584(85.49%) | 91.84% |
| Cse2 | 46897492 | 40712957(86.81%) | 39677161(84.6%) | 92.66% |
| Cse3 | 55185436 | 48434154(87.77%) | 47124875(85.39%) | 91.36% |
| RCsiu1 | 47573810 | 39989350(84.06%) | 37821033(79.5%) | 94.12% |
| RCsiu2 | 54779134 | 45972502(83.92%) | 43467175(79.35%) | 94.31% |
| RCsiu3 | 58493778 | 49262362(84.22%) | 46564950(79.61%) | 94.57% |
| RCseu1 | 58049500 | 48634946(83.78%) | 45905034(79.08%) | 93.87% |
| RCseu2 | 50387400 | 42235712(83.82%) | 39845524(79.08%) | 93.55% |
| RCseu3 | 44461884 | 37013925(83.25%) | 34944793(78.59%) | 94.09% |
| RCsim1 | 53268562 | 44390737(83.33%) | 41936483(78.73%) | 93.64% |
| RCsim2 | 60117918 | 50749749(84.42%) | 47952186(79.76%) | 94.51% |
| RCsim3 | 55854536 | 47034003(84.21%) | 44332177(79.37%) | 94.96% |
| RCsil1 | 56139998 | 45930440(81.81%) | 43334494(77.19%) | 94.79% |
| RCsil2 | 72575920 | 61019493(84.08%) | 57539804(79.28%) | 94.55% |
| RCsil3 | 65949698 | 54952295(83.32%) | 51824893(78.58%) | 95.26% |
| RCsel1 | 56772962 | 47546433(83.75%) | 44869179(79.03%) | 94.46% |
| RCsel2 | 49529880 | 41154451(83.09%) | 38735122(78.21%) | 94.30% |
| RCsel3 | 52472186 | 42972031(81.89%) | 40526389(77.23%) | 92.87% |

1. Csi3 was not showed due to the poor repetition among the three biological replicates.

**Table S2.** Functional categorizations of genes showing ELD in the hybrid

| **Sets** | **Ontology** | **GO terms** | **Padj** |
| --- | --- | --- | --- |
| siu-R-ELD^a^ | MF | phosphoglycerate kinase activity | 2.86E-02 |
|  | MF | phosphotransferase activity, carboxyl group as acceptor | 2.86E-02 |
|  | MF | phosphatidylinositol phospholipase C activity | 2.93E-02 |
|  | MF | phospholipase C activity | 2.93E-02 |
| siu-C-ELD | MF | protein heterodimerization activity | 2.14E-03 |
|  | MF | rRNA binding | 7.92E-03 |
|  | MF | prephenate dehydratase activity | 1.34E-02 |
|  | CC | thylakoid | 2.25E-02 |
| sim-C-ELD | BP | photosynthesis | 2.01E-07 |
|  | CC | thylakoid | 6.33E-07 |
|  | CC | thylakoid part | 6.33E-07 |
|  | CC | photosystem | 1.47E-06 |
| sil-R-ELD | MF | flavin adenine dinucleotide binding | 1.59E-02 |
| sil-C-ELD | MF | protein heterodimerization activity | 1.17E-06 |
|  | MF | prephenate dehydratase activity | 7.23E-03 |
|  | MF | rRNA binding | 1.24E-02 |
|  | BP | L-phenylalanine biosynthetic process | 1.33E-02 |
| seu-R-ELD | CC | large ribosomal subunit | 6.91E-03 |
|  | CC | ribosomal subunit | 6.91E-03 |
|  | MF | ribonuclease T2 activity | 3.93E-02 |
|  | MF | endoribonuclease activity, producing 3'-phosphomonoesters | 3.93E-02 |
| seu-C-ELD | MF | rRNA binding | 2.19E-03 |
|  | MF | protein heterodimerization activity | 2.32E-03 |

1. Only the top four most significantly enriched terms were showed if there were more than four enrichment results. No significant enrichment results for sim-R-ELD.

**Table S3.** Primers used for RT-qPCR

| **Gene** | **Forward primers** | **Reverse primers** |
| --- | --- | --- |
| U6 | GGGGACATCCGATAAAATT | TGTGCGTGTCATCCTTGC |
| 106301024 | CTGGAACGGTATCACTTGC | TTGGCTAACGACGAGGAG |
| 108825747 | CAAAACACAAACTACTACGACCTC | CATAATGGCGGCGAAACC |
| 108814527 | CTCAAACGCAACCCTCAC | ACTTCACGATGGACTCTGG |
| 106342590 | TCTAAGAACTTGACCAACGAG | AGAATCACCATACCCTAAACC |
| 108816675 | GGCTGTTGATTCTTACCGA | TGTGCTTTCCCTTGTTGTT |
| 108838328 | AGAAAAGAAGAATGGAGGCA | CTGTTTGTCGGCAAGTCG |
| 108843528 | GGGAGTGTTGTTGCCATT | GGAGTTGAGCAGCCTTGA |
| 108844774 | ACTGATGACCTCCTCAACTACA | TCACAACTGCCTCCATAAAC |
| 108851476 | AAATGTCGGAACTGAGATGG | CGGAAACGAAGGCAAAAG |
| 106340336 | GAGTCGTTGGATGGATTTCG | TGTTCCGTTCGTTGGTGC |
| 108826229 | AGGTAGCAGCAAAGCGTG | CAAGACCAAGGGCAATGAT |
| 108810935 | GAAAGTGCCCTAAAGATACCC | TGCTTCAACATCAGCGAGT |
